# Tocotrienol Supplementation Led to Higher Serum Levels of Lysophospholipids but Lower Acylcarnitines in Postmenopausal Women: A Randomized Double-Blinded Placebo-Controlled Clinical Trial

**DOI:** 10.3389/fnut.2021.766711

**Published:** 2021-12-24

**Authors:** Chwan-Li Shen, Huanbiao Mo, Dale M. Dunn, Bruce A. Watkins

**Affiliations:** ^1^Department of Pathology, Texas Tech University Health Sciences Center, Lubbock, TX, United States; ^2^Center of Excellence for Integrative Health, Texas Tech University Health Sciences Center, Lubbock, TX, United States; ^3^Center of Excellence for Translational Neuroscience and Therapeutics, Texas Tech University Health Sciences Center, Lubbock, TX, United States; ^4^Nutrition, Georgia State University, Atlanta, GA, United States; ^5^Department of Nutrition, University of California, Davis, Davis, CA, United States

**Keywords:** vitamin E, metabolomics (OMICS), lipids, metabolites, clinical trial, dietary supplement

## Abstract

Osteoporosis is a major health problem in postmenopausal women. Herein we evaluated the effects of 12-week tocotrienols (TT) supplementation on serum metabolites in postmenopausal, osteopenic women. Eighty-nine participants (59.7 ± 6.8 yr, BMI 28.7 ± 5.7 kg/m^2^) were assigned to 3 treatments: placebo (860 mg olive oil/day), 300mg TT (300 mg TT/day), and 600mg TT (600 mg TT/day) for 12 weeks. TT consisted of 90% δ-TT and 10% γ-TT. In this metabolomic study, we evaluated the placebo and 600mgTT at baseline and 12 weeks. As expected, TT and its metabolite levels were higher in the supplemented group after 12 weeks. At baseline, there were no differences in demographic parameters or comprehensive metabolic panels (CMP). Metabolomics analysis of serum samples revealed that 48 biochemicals were higher and 65 were lower in the 600mg TT group at 12 weeks, compared to baseline. The results confirmed higher serum levels of tocotrienols and lysophospholipids, but lower acylcarnitines and catabolites of tryptophan and steroids in subjects given 600mg TT. In summary, 12-week TT supplementation altered many serum metabolite levels in postmenopausal women. The present study supports our previous findings that TT supplementation helps reduce bone loss in postmenopausal osteopenic women by suppressing inflammation and oxidative stress. Furthermore, the body incorporates TT which restructures biomembranes and modifies phospholipid metabolism, a response potentially linked to reduced inflammation and oxidative stress.

## Introduction

Vitamin E contains two subgroups, namely tocopherols and tocotrienols (TT) and their corresponding isomers (α-, β-, γ-, and δ-form), with a common chromanol ring and a saturated or unsaturated side chain (1). Most plants contain tocopherols, while TT can only be found in certain plants (e.g., annatto, palm, grains, and nuts) (2, 3). Compared to tocopherols, the short, unsaturated isoprenoid side chain in TT are more easily incorporated into cell membranes (4). Consequently, the uptake of TT is more rapid than tocopherols (5). In the past decade, TT has gained increased attention because of their potent antioxidant and anti-inflammatory capacities. Among all 8 vitamin E isomers, δ-TT has the greatest antioxidant potency and α-tocopherol has the least potency (6). There is growing evidence that TT protect against the development of age-associated chronic diseases and metabolic derangement, such as obesity (7–9), CVD (10), type-2 diabetes (11, 12), non-alcoholic fatty liver disease (13), arthritis (14–16), and osteoporosis (17).

Recent studies provide strong evidence for age-associated mitochondrial dysfunction, dysregulated intracellular lipid metabolism, and insulin resistance (18), all of which are implicated in the increased incidence of obesity, type 2 diabetes (19), non-alcoholic fatty liver disease (13), and cardiovascular diseases (20) that occur with aging. The mitochondrial electron transport chain is a source for the reactive oxygen species (ROS) and reactive nitrogen species (RNS). Both ROS and RNS are normally generated by highly regulated enzymes such as nitric oxide synthase (NOS) and nicotinamide adenine dinucleotide phosphate (NADPH) oxidase in physiological and pathological conditions (19, 21–23). The chronic overproduction of ROS/RNS, a phenomenon often linked with aging (23), is detrimental to cells and tissues, especially due to ROS/RNS-inflicted damages to key biomolecules for sustaining life, such as lipids, proteins, and DNA (19, 23, 24).

Increased oxidative stress and inflammation in skeletal muscle contributes to structural and functional changes of mitochondria, the important organelle for energy supply, redox regulation, and apoptosis (25). The mitochondrial-protective action of TT is due to antioxidant and anti-inflammatory properties in skeletal muscle, the main organ tightly linked to mitochondrial function. Tocopherols and TT are metabolized by cytochrome P-450 via ω-oxidation, followed by β-oxidation in the mitochondria or by conjugation (26). Most vitamin E, including TT, enters the mitochondrial fractions and in the endoplasmic reticulum due to fat solubility. TT has been known to protect against oxidative stress-induced mitochondrial dysfunction in various organs of animals (27). For example, Sridharan et al. reported a single oral dose of TT-rich fraction (TRF) protects against radiation-induced changes in cardiac mitochondria of rats (27). α-TT and γ-TT, not tocopherol, have been shown to activate peroxisome proliferator-activated receptors α, γ, and δ (PPARα, PPARγ, and PPARδ) and PPAR target genes (28) that play essential roles in energy metabolism, mitochondrial biogenesis, and skeletal muscle fiber distribution (29). Lee et al. demonstrated that relative to the control group, tocotrienol-rich fraction-treated rats had suppressed oxidative stress, enhanced exercise tolerance, and attenuated muscle and liver glycogen use in forced swimming model (30).

Due to the unsaturated isoprenoid side chain and the ability to easily penetrate tissues, some of the biological effects of TT are superior to those of alpha-tocopherol (the most common type of vitamin E with a saturated side chain). Previous studies have shown the osteoprotective effects of tocotrienols on animals with estrogen-deficient bone loss (31–33) as well as those with testosterone-deficient bone loss (34–36). For example, TT supplementation was reported to exert better antioxidant effects in bones than alpha-tocopherol (37) and to improve bone formation in estrogen-deficient rats (38).

Metabolomics profiling technologies provide a systematic approach for evaluating the metabolic response to nutritional interventions by simultaneously measuring and identifying a large number of small molecules. Metabolomics is particularly useful in interpreting human responses to dietary manipulation, including dietary supplement, and improves the capacity to capture both complex and subtle influences on whole body metabolism and physiology. The primary goal of metabolomics is to correlate changes in the biochemical profile of a sample with a corresponding shift in physiology due to the dietary intervention. We recently showed that dietary supplementation with annatto-extracted TT lowered the levels of serum metabolites, dicarboxylic fatty acids, and inflammatory/oxidative stress markers (trimethylamine N-oxide, kynurenate, 12,13-DiHOME, and 13-HODE + 9-HODE) in obese mice, suggesting TT supplementation reduces inflammation and oxidative stress (oxidized glutathione and GSH/GSSH) and improves macronutrient metabolism (carbohydrates) (39).

In the current study, we have translated animal findings to focus research on a clinical trial using postmenopausal women (PMW). In PMW, the rate of bone loss increases dramatically due to estrogen deficiency and contributes to greater bone fractures. Estrogen is a phenolic compound that shares similarities with the structure of well-known lipophilic antioxidants such as α-tocopherol (40). Thus, estrogen has the capacity to detoxify accumulated ROS and estrogen deficiency has been linked to increased oxidative stress (40).

We previously reported that 12-week annatto-extracted TT supplementation decreased bone resorption and improved bone turnover rate via suppressing bone remodeling regulators, namely serum soluble receptor activators of nuclear factor-kappaB ligand and serum osteoprotegerin, in PMW with estrogen deficiency (17). Therefore, this study investigated the metabolic differences between PMW with low bone mass at baseline and after 12 weeks supplementation with 600mg TT. The objective was to examine if dietary annatto-extracted TT supplementation improves macronutrient metabolism in PMW. The body has the mandate to use all nutrients consumed. First, for energy production. Second, as building blocks of cells, tissues, and organs. Lastly, the removal of toxic compounds. It appears that the body incorporates TT to restructure biomembranes that alter phospholipid metabolism. Thus, we hypothesize that annatto-extracted TT affects fatty acids and lysophospholipids levels, and changes macronutrient metabolism as TT is incorporated in cell membranes and tissues.

## Materials and Methods

### Subject Design

This metabolomic study is a subset of a randomized, double-blinded, and placebo-controlled study approved by the Ethical Committee of the Texas Tech University Health Sciences Center, which was registered at ClinicalTrials.gov as NCT02058420. Details of the study design have been published previously (41). Briefly, eighty-nine postmenopausal women with bone densitometry-confirmed osteopenia (mean age 59.7 years, BMI 28.7 kg/m^2^) with no known bone metabolic disease or serious health issues (e.g., history of cancer within the past 5 years, cognitive impairment, severe depression, or medical/eating disorders) were recruited for this study. Participants were randomly assigned into three groups: Placebo, 300mg TT, and 600mg TT for 12 weeks. All participants were provided with dietary calcium (500 mg elemental calcium as calcium carbonate from oyster shell) and vitamin D (400 IU as cholecalciferol, GlaxoSmithKline, PA, USA) daily during the study period. Serum samples were collected at baseline, 6 weeks, and 12 weeks (end) for outcome measures. Eighty-seven subjects completed the study, with two dropouts due to loss of interest. Pill compliance rates for placebo, 300mg TT, and 600mg TT were 93, 92, and 91%, respectively. We have previously reported the flow of subject recruitment, exclusion, dropout, and the final number of subjects in the main study (42). In this metabolomics study, 20 subjects per group, randomly selected from both the Placebo and 600mg TT groups of the larger study (at baseline and 12 weeks), were used for serum metabolomics analyses (Metabolon, Inc., Morrisville, NC, USA).

### Study Treatments

The study supplements (placebo and tocotrienols) have been previously described (17). In brief, the Placebo participants received two 430-mg refined olive pomace oil soft gels per day and the 600mg TT participants received two 430-mg TT soft gels per day, each containing 300 mg TT. TT soft gel was made from DeltaGoldⓇ Annatto Tocotrienol 70%, an extract of annatto seeds containing 90% δ-TT and 10% γ-TT with 70% purity. The majority ingredient in the remaining 30% of TT soft gel was triglyceride. All study soft gels were provided by American River Nutrition, LLC, Hadley, MA. The olive oil soft gels and TT soft gels had no difference in appearance, taste, and smell. All gels were registered with FDA Investigational New Drug (IND) number 120,761.

### Data Collection

Demographics and medical history were obtained at the time of enrollment. Food intake was determined by a semi-quantitative Harvard Willett Food Frequency Questionnaire, physical activity level via Godin Leisure-Time Exercise Questionnaire, and body composition by SC-331S Body Composition Analyzer (Tanita Corporation of America, Inc., Arlington Height, IL, USA) were recorded at baseline and after 12 weeks. Overnight fasting blood was drawn from a superficial arm vein, and serum samples were collected after centrifugation (1500 × *g* for 20 min), and stored in −80°C freezer before analysis.

### Metabolomics Analyses of Serum

Non-targeted Metabolomics analysis of biochemicals in serum samples was conducted at Metabolon, Inc (Morrisville, NC). Sample preparation was carried out in a manner similar to a previous study (43). In brief, individual samples were subjected to methanol extraction according to Evans et al., then reconstituted and split into aliquots for analysis by ultrahigh-performance liquid chromatography/mass spectrometry (UHPLC/MS) (44). The global biochemical profiling analysis was comprised of four unique arms consisting of: reverse phase chromatography positive ionization methods optimized for hydrophilic compounds (LC/MS Pos Polar) and hydrophobic compounds (LC/MS Pos Lipid), reverse phase chromatography with negative ionization conditions (LC/MS Neg), as well as a HILIC chromatography method coupled to negative (LC/MS Polar) (45). All of the methods alternated between full scan MS and data dependent MS^*n*^ scans. The scan range varied slightly between methods but generally covered 70–1000 *m*/*z*.

Metabolites were identified by automated comparison of the ion features in the experimental samples to a reference library of chemical standard entries that included retention time, molecular weight (m/z), preferred adducts, and in-source fragments as well as associated MS spectra and curated by visual inspection for quality control using software developed at Metabolon. Identification of known chemical entities was based on comparison to metabolomic library entries of purified standards (46). The identification also included KEGG pathway database analysis, especially in figures illustrating specific metabolism of metabolites.

### Statistical Analyses

Two types of statistical analyses for metabolites were performed: (1) significance tests and (2) classification analysis. Standard statistical analyses were performed in ArrayStudio on log-transformed data. For analyses not standard in ArrayStudio, the R program (http://cran.r-project.org/) was used. For statistical analyses and data presentation, biochemicals were rescaled to set the median to one, based on all samples. Following log transformation and imputation of missing values, any missing values were assumed to be below the limits of detection and were imputed with the compound minimum (minimum value imputation). As the minimum observed value for each compound, ANOVA contrasts were used to identify biochemicals that differed significantly between experimental groups.

To detect differences for the metabolites in serum, mixed model ANOVA (main effect: treatment and time, random effect: subject ID) a standard statistical test completed with R program (http://cran.r-project.org/) was used to compare between the placebo- and High TT-treated groups at baseline and after 12 weeks. *P*-values <0.05 were considered significant. We further performed Welch's *t*-test, a standard statistical test completed with R program (http://cran.r-project.org/), for ratio High-TT (12 weeks vs. 0 week) to the ratio Placebo (12 weeks vs. 0 week) to identify biochemicals that differed significantly (*p* < 0.05) between experimental groups. An estimate of the false discovery rate (*q*-value) was calculated to account for the multiple comparisons that normally occur in metabolomic-based studies. Classification analyses used included principal components analysis (PCA), hierarchical clustering, and random forest. Analyses were graphed using Array Studio software, version 7.2 (Omicsoft, NC). The Random forest is a supervised classification technique based on an ensemble of decision trees (47). For the scaled intensity graphics, each biochemical in original scale (raw area count) was rescaled to set the median across all subjects and time-points equal to 1. All values are presented as ratios fold of change (least squares means), placebo 12 weeks/placebo 0 weeks, and High-TT 12 weeks/High-TT 0 weeks.

## Results

### Study Population

Herein 416 postmenopausal women aged 40 years and older were recruited to participate in this study, and provided informed consent. In total, 326 subjects were excluded from the study due to not meeting inclusion criteria (*n* = 234), decline to participate (*n* = 80), and other reasons (*n* = 12). The remaining 89 subjects were randomized into three groups (Placebo, 300mg TT, and 600mg TT) and 87 subjects completed the study (attrition rate: 2%) with a >90% compliance rate by a pill count. In this metabolic profiling study, only serum samples from Placebo and 600mg TT groups at baseline and after 12 weeks were performed. Participants in the Placebo and 600mg TT groups showing no significant differences in age (60.8 ± 5.2 yr for Placebo vs. 59.2 ± 6.6 yr for 600mg TT), weight (71.8 ± 12.6 kg for Placebo vs. 71.9 ± 14.8 kg for 600mg TT), height (158.8 ± 6.1 cm for Placebo vs. 159.2 ± 5.9 cm for 600mg TT), and BMI (28.5 ± 4.9 kg/m^2^ for Placebo vs. 28.2 ± 5.5 kg/m^2^ for 600mg TT). We did not find significant changes in (a) macro- and micronutrients intakes, (b) physical activity level, and (c) body composition between the Placebo and the 600mg TT groups at both baseline and at the end of study (*p* > 0.05). The detailed report of medical history, macro- and micro-nutrients intake, physical activity, and body composition of study participants has been published previously (48).

### Metabolite Levels in Subjects

A comprehensive non-targeted mass spectrometry-based metabolomics profiling analysis was performed on sera from the placebo and 600mg TT groups at baseline and after 12 weeks. Overall, a total of 767 structurally named biochemicals (known metabolites) were detected and categorized into six major categories, namely: amino acids, cofactors and vitamins, lipids, nucleotides, peptides, and xenobiotics.

**Figure 1:**
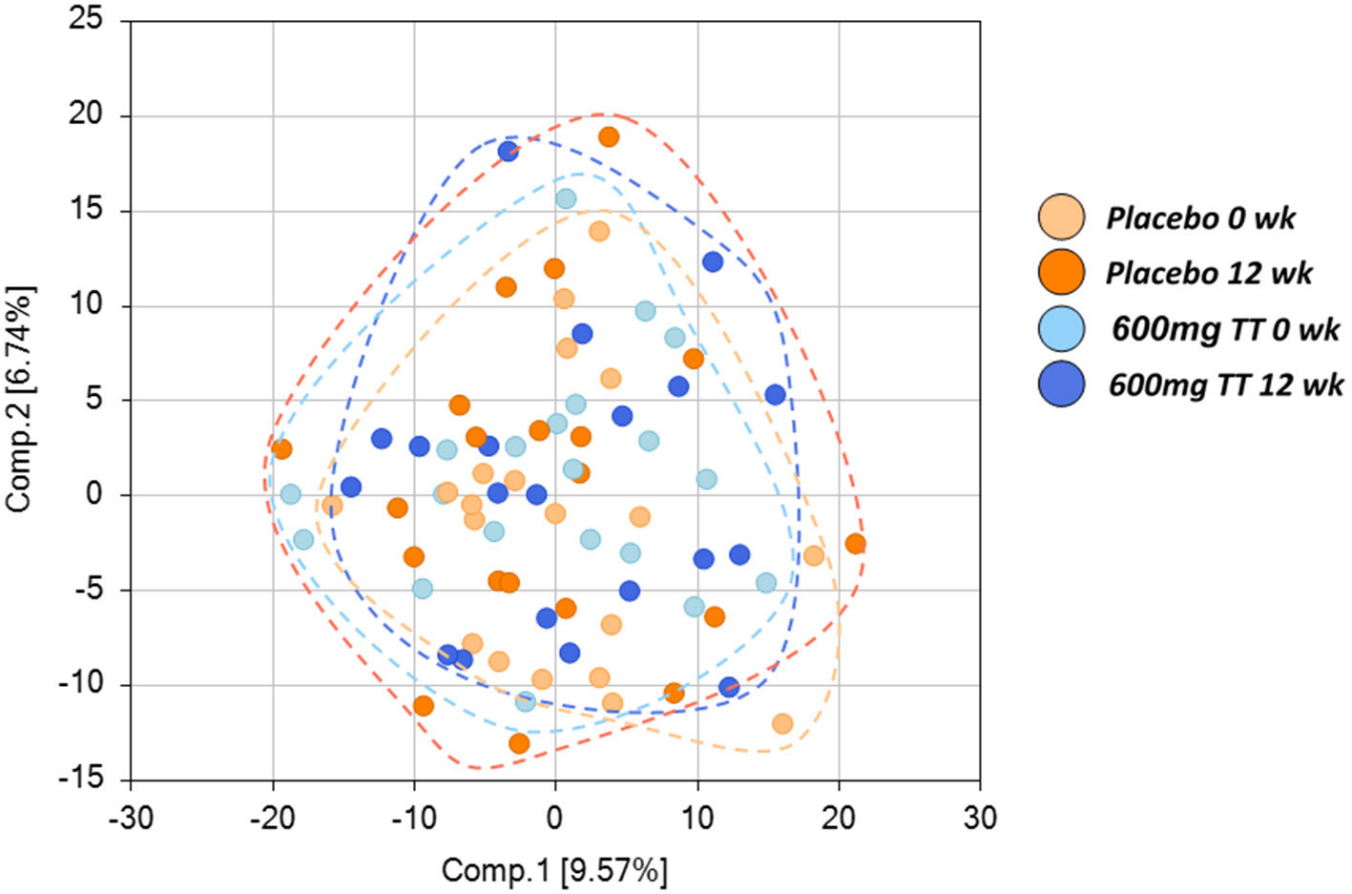
Score plot of principle component analysis (PCA) of the Placebo and the High TT groups at 0 and 12 wk. Placebo at 0 wk (light orange), Placebo at 12 wk (dark orange), 600 mg TT at 0 wk (light blue), 600mg TT at 12 wk (dark blue). Principal component analysis (PCA) showed no differences in serum metabolites between the Placebo and the 600mg TT group, as demonstrated by the lack of separation between the Placebo and the 600mg TT group. Each ball represents the cumulative metabolites from each participant. The 20 light orange balls are from Placebo group at 0 wk, 20 orange balls are from Placebo group at 12 wk, 20 light blue balls are from 600mg TT group at 0 wk, and 20 dark blue balls are from 600mg TT group at 12 wk.

Among 767 compounds, there were 25 biochemicals primarily responsible for the observed difference due to treatment; 109 due to time effect; and 92 due to the interaction between treatment and time (*p* < 0.05). Principal component analysis (PCA) for serum revealed no separation between the Placebo and the 600mg TT groups (Figure 1). Then, we undertook ANOVA contrasts to identify metabolites that differed significantly between the Placebo and the 600mg TT groups at different times. The results of analysis by two-way ANOVA with repeated measures identified metabolites of 87 (43 higher and 44 lower), 113 (48 higher and 65 lower), 26 (14 higher and 12 lower), and 45 (18 higher and 27 lower) for ratios of Placebo 12wk to Placebo 0wk, 600mg TT 12wk to 600mg TT 0 wk, 600mg TT 0wk to Placebo 0wk, and 600mg TT 12wk to Placebo 12wk, respectively at *p* ≤ 0.05, exhibiting significant main effects and interactions for experimental parameters of treatment and time point.

### Tocotrienols in Serum

**Figure 2:**
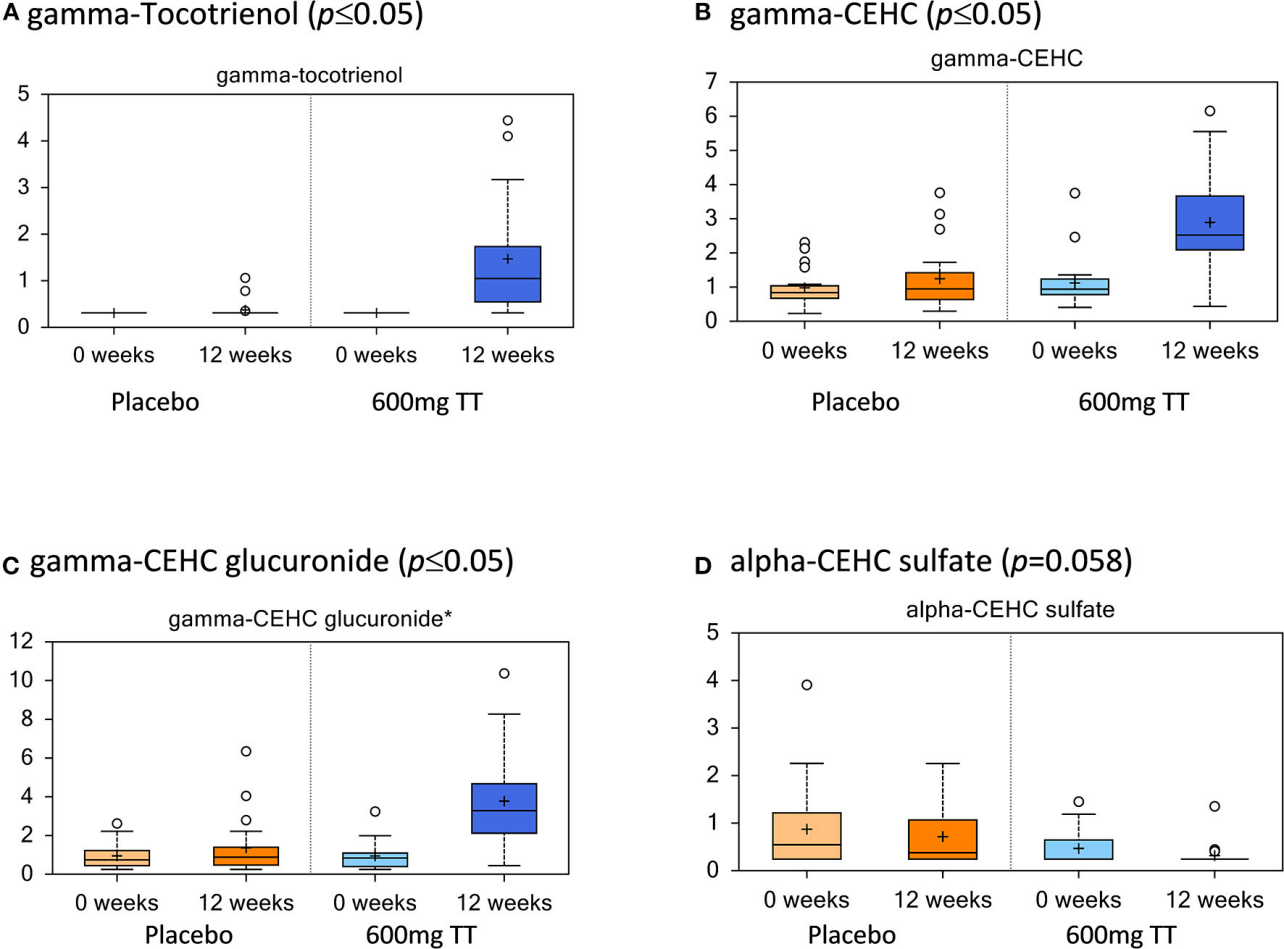
Tocotrienols and their metabolites in serum of the placebo and the 600mg TT groups at 0 and 12 wk. Values represent fold differences in serum tocotrienol and metabolites in placebo and 600mg TT treatment from serum analysis of subjects. Tocotrienols increased with supplementation and were higher compared to the placebo group **(A–D)**.

The levels of serum TT and their metabolites are listed in Figure 2. In agreement with the dietary TT supplementation, increased levels of TT were detected in the treatment cohort at 12 weeks (600mg TT). Gamma-tocotrienol levels were below the detection limit at the beginning of the study (Placebo and 600mg TT), whereas at the end of the 12-week study this compound was detected in serum from 18 women consuming TT and only 4 women from placebo group. Based on the existing Metabolon compound library, gamma-tocotrienol was the only TT detected in serum. Also, gamma-tocotrienol metabolites, gamma-2-(beta-carboxyethyl)-6-hydroxy-2,7,8-trimethylchroman (gamma-CEHC, the terminal beta-oxidation product of gamma-tocotrienol), and gamma-CEHC glucuronide (the glucuronidated form of gamma-CEHC) were significantly elevated in the 600mg TT group after 12 weeks. The levels of alpha-CEHC sulfate, an alpha TT metabolite, showed a reduced trend in the 600mg TT group vs. baseline (*p* = 0.058) and significantly lower in the 600mg TT group vs. Placebo at 12 weeks of treatment.

### Metabolites Associated With Bone and Collagen

The changes in metabolites associated with bone matrix are listed in Table 1.

**Table 1:** Changes in metabolites associated with bone matrix in serum of the Placebo and the 600mg TT groups at 0 and 12 wk.

**Sub pathway**	**Biochemical name**	**Placebo:** **Ratio 12 wk/0 wk**	**600mg TT:** **Ratio 12 wk/0 wk**
Tryptophan metabolism	C-glycosyltryptophan	1.08^‡^	0.93^‡^
Urea cycle; arginine and proline metabolism	Proline-hydroxyproline (pro-hydroxy-pro)	1.03	0.82^*^
Amino sugar metabolism	N-acetylglucosamine/N-acetylgalactosamine	1.06	0.89^‡^
	Glucuronate	0.96	1.25^*^

*Metabolite values are expressed as the ratio of Placebo 12 wk/0 wk which is the fold change of the Placebo at 12 wk compared to the Placebo at 0 wk. Similarly, the ratio of 600mg TT 12 wk/0 wk which is the fold change of the 600mg TT at 12 wk compared to the High TT at 0 week. A ratio >1 indicates a value larger for the 12-wk collection (Placebo 12 wk or High TT 12 wk) and <1 the value for the 12-wk collection (Placebo 12 wk or 600mg TT 12 wk) is lower compared to the 0-wk collection (Placebo 0 wk or 600mg TT 0 wk). Statistical differences are indicated for the values at baseline and after 12 weeks within the same treatment group or placebo (n = 20)*.

* *Indicates p < 0.05*.

‡ *Indicates 0.05 < p < 0.1*.

Compared to the 600mg TT group at 0 week, both of C-glycosyltryptophan (*p* = 0.081) and proline-hydroxyproline (pro-hydroxy-pro) (*p* = 0.0003) were lower after 12 weeks of TT supplementation. Furthermore, there was a trend toward a lower level in N-acetylglucosamine/N-acetylgalactosamine (measured as an isobar, *p* = 0.09) and higher serum glucuronate levels (*p* < 0.05) in the 600mg TT groups from 0 week to 12 weeks.

### Changes in Metabolites Associated With Redox Environment

**Figure 3:**
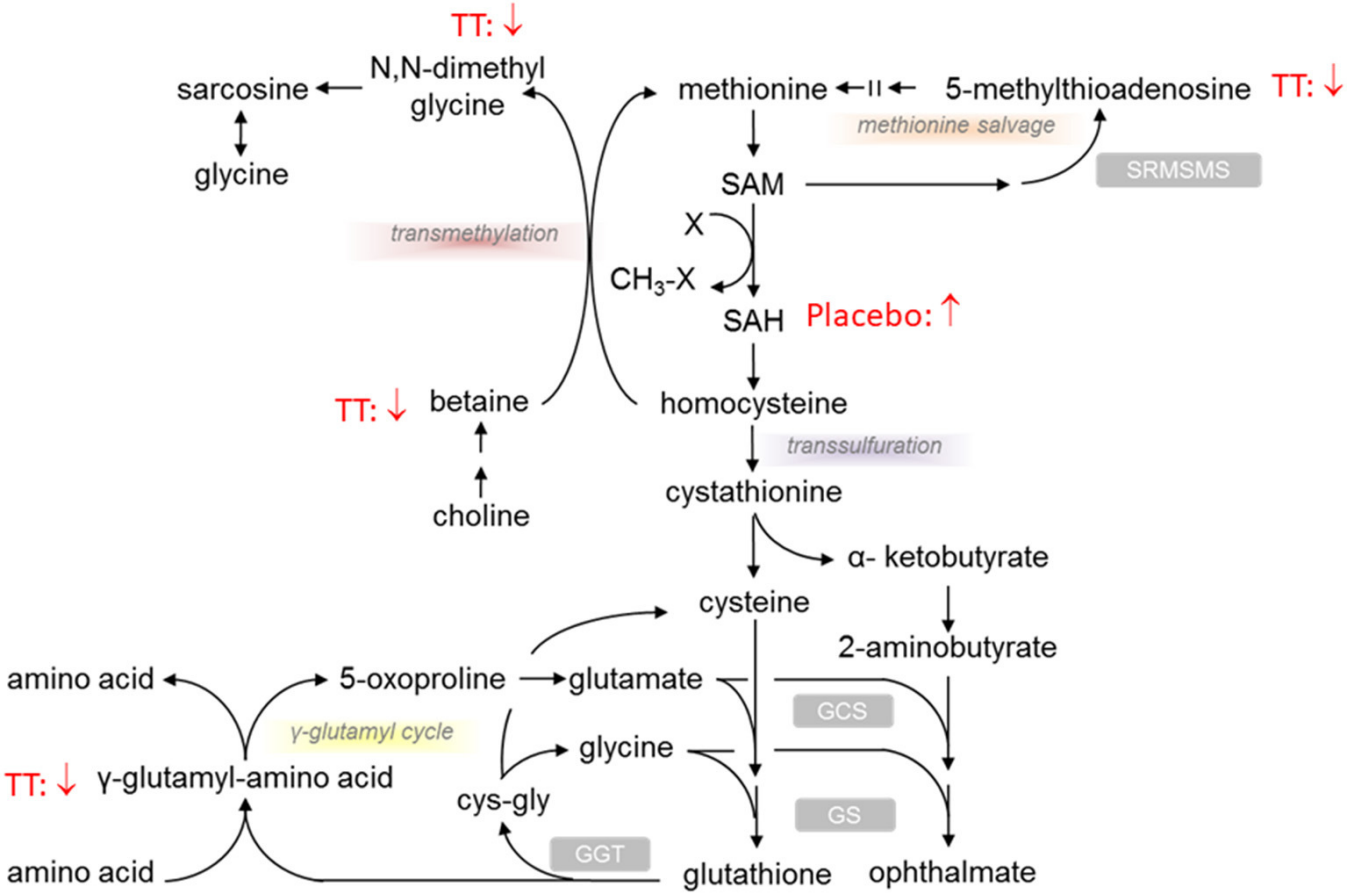
Effect of tocotrienols supplementation on redox environment biochemical pathways. Proposed actions of tocotrienols on compounds associated with redox pathways based on serum analysis from subjects. Tocotrienols tended to result in lower levels of compounds where the downward arrows are shown.

Significant differences were noted in transmethylation and transsulfuration pathway biochemicals with lower levels of dimethylglycine, betaine, and 5-methylthioadenosine (MTA), when comparing serum levels after 12 weeks of TT administration (600mg TT after 12 weeks vs. 600mg TT baseline) as shown in Table 2. Compared to the baseline for the 600mg TT group, after 12 weeks of TT supplementation, the levels of gamma-glutamyl amino acid metabolites (e.g., gamma-glutamyltyrosine, gamma-glutamylmethionine, gamma-glutamylleucine) were lower in the serum of TT-supplemented subjects (Table 2). Most of the metabolites of methionine and cysteine (e.g., methionine, N-acetylmethionine, N-formylmethionine, N-acetylmethionine sulfoxide, and S-adenosylhomocysteine) were lower in the 600mg TT groups after 12 weeks of intervention (all *p* < 005). In this case TT supplementation may conserve met and cys amino acids, sulfur derivatives or lead to a lower catabolism of these amino acids. Furthermore, the sulfur containing amino acids (Figure 3) such as methionine and cysteine are more susceptible to oxidation by ROS and appear to be protected by supplemental TT (49).

**Table 2:** Changes in metabolites associated with redox environment in serum of the Placebo and the 600mg TT groups at 0 and 12 wk.

**Sub pathway**	**Biochemical name**	**Placebo:** **Ratio 12 wk/0 wk**	**600mg TT:** **Ratio 12 wk/0 wk**
Glycine, serine, and threonine metabolism	Dimethylglycine Betaine	0.99 1.01	0.91^*^ 0.92^*^
Polyamine metabolism	5-methylthioadenosine (MTA)	1.03	0.93^*^
Gamma-glutamyl amino acid	Gamma-glutamylleucine Gamma-glutamylmethionine Gamma-glutamyltyrosine	1.04 1.00 1.01	0.88^*^ 0.89^*^ 0.83^*^
Methionine, cysteine, SAM, and taurine metabolism	Methionine N-acetylmethionine N-formylmethionine Methionine sulfone N-acetylmethionine sulfoxide S-adenosylhomocysteine (SAH) Cystathionine Cysteine sulfinic acid	1.01 1.26^*^ 1.07^*^ 0.96 1.38^*^ 1.26^*^ 1.53^*^ 1.10	0.93^*^ 0.98 0.91^*^ 1.15^*^ 0.77^*^ 0.94 1.10 1.12^*^

*Metabolite values are expressed as the ratio of Placebo 12 wk/0 wk which is the fold change of the Placebo at 12 wk compared to the Placebo at 0 wk. Similarly, the ratio of 600mg TT 12 wk/0 wk which is the fold change of the High TT at 12 wk compared to the High TT at 0 week. A ratio >1 indicates a value larger for the 12-wk collection (Placebo 12 wk or 600mg TT 12 wk) and <1 the value for the 12-week collection (Placebo 12 wk or 600mg TT 12 wk) is lower compared to the 0-wk collection (Placebo 0 wk or 600mg TT 0 wk). Statistical differences are indicated for the values at baseline and after 12 weeks within the same treatment group or placebo (n = 20)*.

* *Indicates p < 0.05*.

### Alterations in Tyrosine and Tryptophan Metabolite Levels

Tyrosine and tryptophan metabolites were altered after TT supplementation (Table 3). Changes in tyrosine levels were associated with lower levels in tyrosine, n-acetyltyrosine, 4-hydroxyphenylpyruvate, 3-(4-hydroxyphenyl)lactate (HPLA) and vanillactate, and a higher p-cresol-glucuronide in subjects after 600mg TT administration compared to baseline. Also, HPLA, vanillactate, and p-cresol-glucuronide were higher in the Placebo group after 12 weeks as shown in Table 3.

**Table 3:** Changes in metabolites associated with tyrosine and tryptophan metabolism in serum of the Placebo and the 600mg TT groups at 0 and 12 wk.

**Sub pathway**	**Biochemical name**	**Placebo:** **Ratio 12 wk/0 wk**	**600mg TT:** **Ratio 12 wk/0 wk**
Tyrosine	Tyrosine	1.03	0.92^*^
metabolism	n-Acetyltyrosine	1.03	0.82^*^
	4-Hydroxyphenylpyruvate	1.07	0.81^*^
	3-(4-hydroxyphenyl)lactate (HPLA)	1.16^*^	0.90^*^
	Vanillactate	1.39^*^	0.79
	p-Cresol-glucuronide	1.90^*^	1.72^*^
Tryptophan	n-acetyltryptophan	1.04	0.79^*^
metabolism	Kynurenine	1.00	0.88^*^
	3-indoxyl sulfate	1.15	1.28^*^
Nicotinate and nicotinamide metabolism	Quinolinate	0.94	0.81^*^

*Metabolite values are expressed as the ratio of Placebo 12 wk/0 wk which is the fold change of the Placebo at 12 wk compared to the Placebo at 0 wk. Similarly, the ratio of 600mg TT 12 wk/0 wk which is the fold change of the 600mg TT at 12 wk compared to the High TT at 0 week. A ratio >1 indicates a value larger for the 12-wk collection (Placebo 12 wk or 600mg TT 12 wk) and <1 the value for the 12-wk collection (Placebo 12 wk or 600mg TT 12 wk) is lower compared to the 0-wk collection (Placebo 0 wk or 600mg TT 0 wk). Statistical differences are indicated for the values at baseline and after 12 weeks within the same treatment group or placebo (n = 20)*.

* *Indicates p < 0.05*.

**Figure 4:**
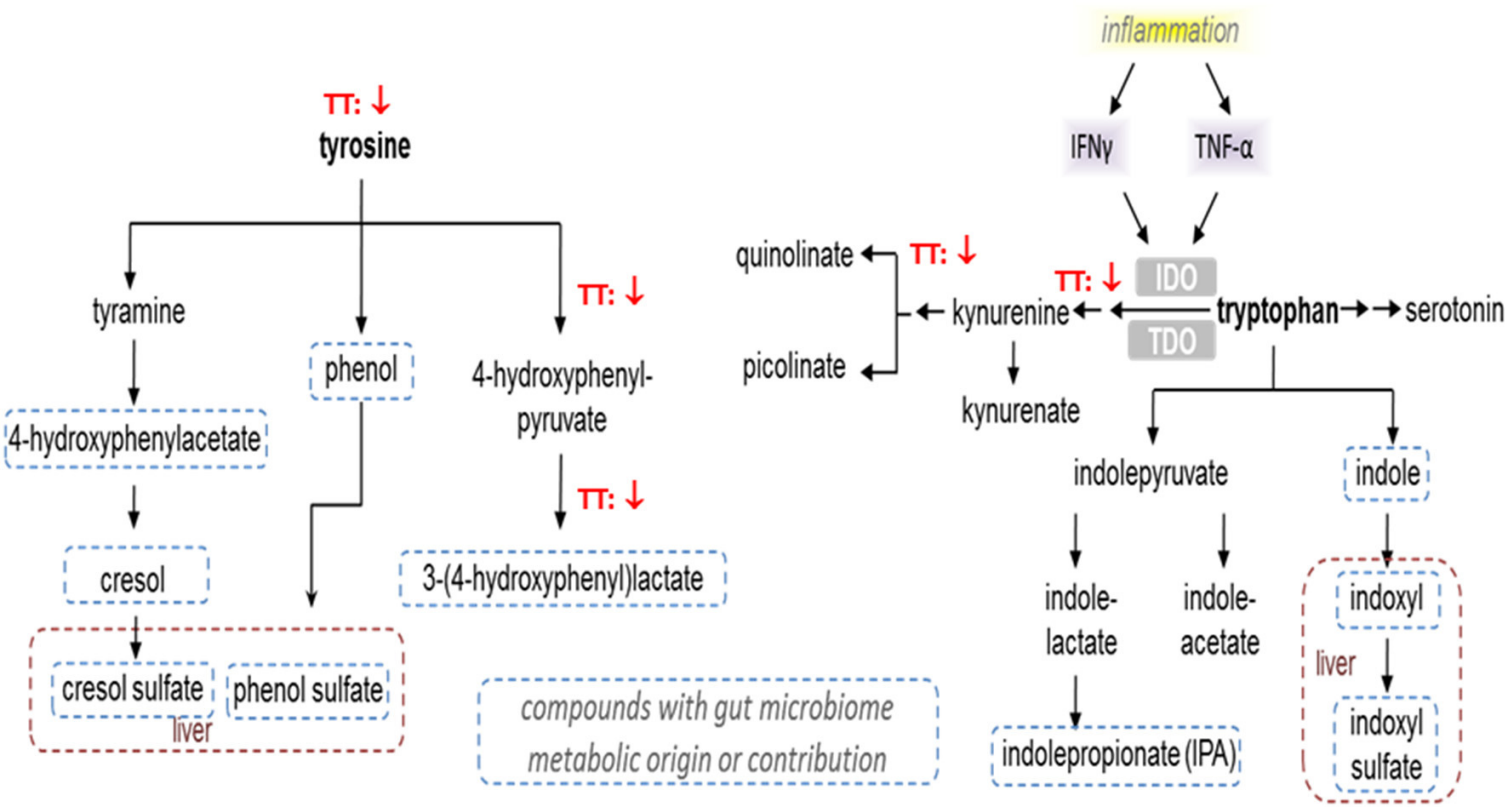
Effect of tocotrienols supplementation on tyrosine and tryptophan metabolism. Proposed actions of tocotrienols on tyrosine and tryptophan metabolites based on serum analysis from subjects. Tocotrienols tended to result in lower levels of compounds where the downward arrows are shown.

In terms of tryptophan metabolites, the subjects given 600 TT for 12 weeks had lower levels of n-acetyltryptophan and kynurenine, while a higher level of 3-indoxyl sulfate (Table 3). Similarly, after 12 week of TT intervention, subjects had decreased serum level of quinolinate (a metabolite involved in nicotinate and nicotinamide metabolism) (Table 3). Under TT supplementation catabolism of essential amino acids (tryptophan) may be reduced thus conserving amino acids (tyrosine and tryptophan) for protein synthesis (Figure 4).

### Changes in Steroid Hormone Metabolites

**Figure 5:**
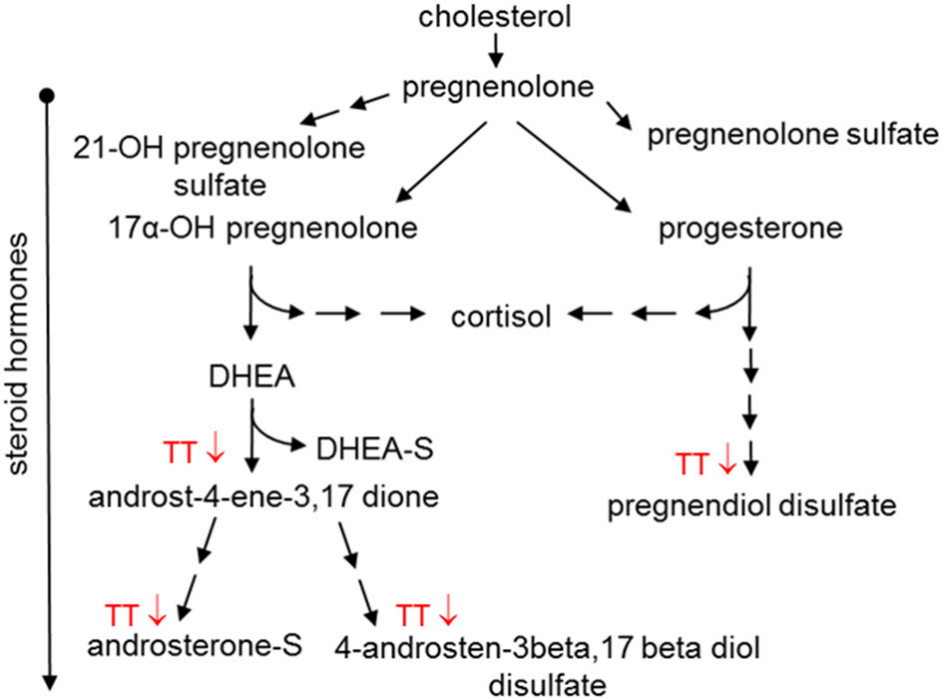
Effect of tocotrienols supplementation on steroid hormone metabolism. Proposed actions of tocotrienols on cholesterol metabolites based on serum analysis from subjects. Tocotrienols tended to result in lower amounts of compounds where the downward arrows are shown.

Steroid hormone metabolism was affected in the Placebo group and to a greater extent in the 600mg TT group after 12 weeks of the study (Table 4). When compared to baseline, the levels of several steroid hormone metabolites (e.g., 5alpha-pregnan-3beta, 20beta-diol monosulfate (1), 5alpha-pregnan-3beta,20alpha-diol disulfate, pregnen-diol disulfate, androsterone sulfate, androstenediol (3alpha, 17alpha) monosulfate (3), androstenediol (3beta, 17beta) disulfate (1), and a series of androstan-diol monosulfate and disulfates) were lower in the 600mg TT group after 12 weeks of intervention. Furthermore, the levels of some metabolites (i.e., pregnenolone sulfate, pregn steroid monosulfate, and dehydroisoandrosterone sulfate (DHEA-S) were lower in Placebo after 12 weeks (Table 4). Thus, changes in steroid hormones after TT supplementation may suggest effects on DHEA and progesterone levels by influencing the expression of biosynthetic enzymes (Figure 5).

**Table 4:** Changes in metabolites associated with steroid hormone metabolism in serum of the Placebo and the 600mg TT groups at 0 and 12 wk.

**Sub pathway**	**Biochemical name**	**Placebo:** **Ratio 12 wk/0 wk**	**600mg TT:** **Ratio 12 wk/0 wk**
Sterol	Cholesterol	0.93^‡^	1.08^‡^
	Beta-sitosterol	0.97	1.25^*^
Steroid	Pregnenolone sulfate	0.76^*^	0.94
	5alpha-pregnan-3beta, 20beta-diol monosulfate (1)	0.97	0.83^*^
	5alpha-pregnan-3beta, 20alpha-diol disulfate	0.93	0.68^*^
	Pregnen-diol disulfate^*^	0.92	0.81^*^
	Pregn steroid monosulfate^*^	0.85^*^	0.98
	Dehydroisoandrosterone sulfate (DHEA-S)	0.88^*^	1.01
	Androsterone sulfate	0.88^‡^	0.82^*^
	Androstenediol (3alpha, 17alpha) monosulfate (3)	0.82^*^	0.82^*^
	Androstenediol (3beta,17beta) disulfate (1)	0.98	0.89^*^
	Androstenediol (3beta,17beta) disulfate (2)	0.93	0.85^‡^
	5alpha-androstan-3alpha, 17beta-diol monosulfate (1)	0.99	0.80^*^
	5alpha-androstan-3beta, 17beta-diol monosulfate (2)	1.05	0.76^*^
	5alpha-androstan-3beta,17alpha-diol disulfate	0.89	0.60^*^
	5alpha-androstan-3alpha,17beta-diol disulfate	0.88	0.60^*^
	5alpha-androstan-3beta, 17beta-diol disulfate	1.02	0.67^*^

*Metabolite values are expressed as the ratio of Placebo 12 wk/0 wk which is the fold change of the Placebo at 12 wk compared to the Placebo at 0 wk. Similarly, the ratio of 600mg TT 12 wk/0 wk which is the fold change of the 600mg TT at 12 wk compared to the 600mg TT at 0 week. A ratio >1 indicates a value larger for the 12-wk collection (Placebo 12 wk or 600mg TT 12 wk) and <1 the value for the 12-wk collection (Placebo 12 wk or 600mg TT 12 wk) is lower compared to the 0-wk collection (Placebo 0 wk or High TT 0 wk). Statistical differences are indicated for the values at baseline and after 12 weeks within the same treatment group or placebo (n = 20)*.

* *Indicates p < 0.05*.

‡ *Indicates 0.05 < p < 0.1*.

### Changes in Levels of Lysophospholipids

Compared to baseline, the levels of several serum lysophospholipids were higher in individuals consuming TT for 12 weeks, e.g., 2-palmitoyl-glycerophosphocholine (GPC), 1-palmitoleoyl-GPC, 2-palmitoleoyl-GPC, 1-stearoyl-GPC, 1-oleoyl-GPC, 1-linoleoyl-GPC, and 1-arachidonoyl-GPC, 1 palmitoyl- glycosylphosphatidylinositol (GPI), 1-stearoyl-GPI, 1-oleoyl-GPI, 1-linoleoyl-GPI, and 1-archidonoyl-GPI (Table 5). In contrast, only the Placebo group showed higher levels of other lysophospholipid metabolites, such as 1-linolenoyl-GPC, 1-stearoyl-glycerophosphoethanolamine (GPE), 1-oleoyl-GPE, and 1-linoleoyl-GPE at the study period end (Table 5). Although the levels of some lysophospholipids (expressed as ratio at baseline to 12 weeks) were observed in the Placebo group, in general TT resulted in large differences in these bioactive signaling lipids that are generated from phospholipase-mediated hydrolyzation of membrane lipids.

**Table 5:** Changes in metabolites associated lysophospholipids in serum of the Placebo and the 600mg TT groups at 0 and 12 wk.

**Sub pathway**	**Biochemical name**	**Placebo:** **Ratio 12 wk/0 wk**	**600mg TT:** **Ratio 12 wk/0 wk**
Lysophospholipids	2-palmitoyl-GPC (16:0)	1.00	1.17^*^
	1-palmitoleoyl- GPC (16:1)	1.06	1.20^*^
	2-palmitoleoyl- GPC (16:1)	1.13	1.24^*^
	1-stearoyl-GPC (18:0)	1.09^*^	1.09^*^
	1-oleoyl-GPC (18:1)	1.09^*^	1.13^*^
	1-linolenoyl-GPC (18:3)	1.37^*^	1.16
	1-arachidonoyl- GPC (20:4n6)	1.03	1.07^*^
	1-stearoyl-GPE (18:0)	1.12^*^	1.07
	1-oleoyl-GPE (18:1)	1.21^*^	1.05
	1-linoleoyl-GPE (18:2)	1.15^*^	0.98
	1-palmitoyl-GPI (16:0)	1.04	1.57^*^
	1-stearoyl-GPI (18:0)	1.05	1.46^*^
	1-oleoyl-GPI (18:1)	1.19	1.64^*^
	1-linoleoyl-GPI (18:2)	1.01	1.24^*^
	1-arachidonoyl- GPI (20:4)	0.94	1.29^*^

*Metabolite values are expressed as the ratio of Placebo 12 wk/0 wk which is the fold change of the Placebo at 12 wk compared to the Placebo at 0 wk. Similarly, the ratio of 600mg TT 12 wk/0 wk which is the fold change of the 600mg TT at 12 wk compared to the 600mg TT at 0 week. A ratio >1 indicates a value larger for the 12-wk collection (Placebo 12 wk or 600mg TT 12 wk) and <1 the value for the 12-wk collection (Placebo 12 wk or 600mg TT 12 wk) is lower compared to the 0-wk collection (Placebo 0 wk or 600mg TT 0 wk). Statistical differences are indicated for the values at baseline and after 12 weeks within the same treatment group or placebo (n = 20)*.

* *Indicates p < 0.05. GPC, glycerophosphocholine; GPE, glycerophosphoethanolamine; GPI, glycosylphosphatidylinositol*.

### Changes in Fatty Acids and Related Biochemicals

The levels of several acyl carnitine metabolites (e.g., acetylcarnitine, cis-4-decenoylcarnitine, linoleolycarnitine, docosahexaenoylcarnitine, linoceroylcarnitine, cerotoylcarnitine, and 3,4-methyleneheptanoylcarnitine) were lower only in the serum samples of subjects consuming TT group at 12 weeks compared to baseline as shown in Table 6. Levels of several saturated (e.g., myristate, pentadecanoate, palmitate, and margarate) and monounsaturated fatty acids (palmitoleate, 10-heptadecenoate, oleate/vaccinate, 10-nonadecenoate, eicosenoate, and erucate) were lower in the Placebo group after the 12-week study period. In terms of polyunsaturated fatty acid metabolites, at the end of intervention, the levels of hexadecadienoate, linoleate, dihomo-linoeate, adrenate, and docosapentaenoate were lower in the Placebo group when compared to those in the TT-supplemented group. Moreover, TT supplementation for 12 weeks resulted in higher docosapentaenoate and dihomo-linolenate compared to baseline. The changes in the levels of fatty acids and related biochemicals is likely due to metabolism and membrane phospholipids incorporating TT in biomembranes and its subsequent antioxidant capacity to protect PUFA (50).

**Table 6:** Changes in metabolites associated fatty acids in serum of the Placebo and the 600mg TT groups at 0 and 12 wk.

**Sub pathway**	**Biochemical name**	**Placebo:** **Ratio 12 wk/0 wk**	**600mg TT:** **Ratio 12 wk/0 wk**
Fatty acid	Acetylcarnitine (2 carbon)	0.95	0.89^*^
metabolism (Acyl Carnitine)	Cis-4-decenoylcarnitine (10:1)	0.89	0.83^*^
	Linoleoylcarnitine (18:2)	0.98	0.81^*^
	Docosahexaenoylcarnitine (22:6)	0.81	0.66^*^
	Lignoceroylcarnitine (24 carbon)	0.95	0.78^*^
	Cerotoylcarnitine (26 carbon)	0.90	0.84^*^
	3,4-methyleneheptanoylcarnitine	0.97	0.48^*^
Saturated and	Myristate (14:0)	0.80^*^	1.16
monounsaturated	Pentadecanoate (15:0)	0.82^*^	1.09
fatty acid	Palmitate (16:0)	0.84^*^	1.05
	Margarate (17:0)	0.81^*^	1.07
	Palmitoleate (16:1n7)	0.69^*^	1.17
	10-heptadecenoate (17:1n7)	0.73^*^	1.17
	Oleate/vaccenate (18:1)	0.79^*^	1.07
	10-nonadecenoate (19:1n9)	0.77^*^	1.05
	Eicosenoate (20:1)	0.77^*^	1.02
	Erucate (22:1n9)	0.84^*^	1.02
Polyunsaturated fatty acid	Hexadecadienoate (16:2n6)	0.69^*^	1.28
(n-6 and n-3)	Linoleate (18:2n6)	0.80^*^	1.05
	Dihomo-linoleate (20:2n6)	0.80^*^	1.05
	Adrenate (22:4n6)	0.78^*^	1.02
	Docosapentaenoate (n6 DPA; 22:5n6)	0.93	1.15^*^
	Dihomo-linolenate (20:3n3 or n6)	0.95	1.17^*^
	Docosapentaenoate (n3 DPA; 22:5n3)	0.83^*^	1.13
	Docosahexaenoate (DHA; 22:6n3)	0.86^‡^	1.11

*Metabolite values are expressed as the ratio of Placebo 12 wk/0 wk which is the fold change of the Placebo at 12 wk compared to the Placebo at 0 wk. Similarly, the ratio of 600mg TT 12 wk/0 wk which is the fold change of the 600mg TT at 12 wk compared to the 600mg TT at 0 week. A ratio >1 indicates a value larger for the 12-wk collection (Placebo 12 wk or 600mg TT 12 wk) and <1 the value for the 12-week collection (Placebo 12 wk or 600mg TT 12 wk) is lower compared to the 0-wk collection (Placebo 0 wk or 600mg TT 0 wk). Statistical differences are indicated for the values at baseline and after 12 weeks within the same treatment group or placebo (n = 20)*.

* *Indicates p < 0.05*.

‡ *Indicates 0.05 < p < 0.1*.

## Discussion

This study is the first double-blinded placebo-controlled randomized study to evaluate the effect of dietary annatto-extracted TT supplement on serum metabolites in postmenopausal women using a non-targeted metabolomics analysis. Tocopherols can be metabolized to carboxyethyl-hydroxychroman (CEHC) derivatives and excreted in the urine (51). CEHC are more hydrophilic than their parent tocopherols, but retain antioxidant properties associated with the hydroxyl group of the chroman ring (52). TT is metabolized essentially the same way as tocopherols through beta-oxidation to CEHC but TT yields higher carboxymethylbutyl hydroxychroman (CMBHC), the second to last product before CEHC. In urine, the level of CMBHC is lower than that of CEHC, probably due to their lipophilic nature. Therefore, more CMBHC is excreted through bile rather than urine (53).

The findings that TT supplementation resulted in the higher levels of gamma-TT, gamma-CEHC (the terminal beta-oxidation metabolites), and gamma-CEHC glucuronide (a gamma-CEHC metabolite) in the serum of subjects corroborates with findings of previous studies (54, 55). Freiser and Jiang reported that gamma-TT is primarily metabolized to conjugated long-chain gamma-CEHC in the plasma of rats (54). Uchida et al. further demonstrated that a single dose of oral TT mixtures (alpha- and gamma-TT) is catabolized in liver to gamma-CEHC, which appears in the serum of rats 24 h after administration (55). In the present study, we noted that the levels of alpha-CEHC sulfate, an alpha TT metabolite, showed a downward trend in 600mg TT at 12 weeks compared to the baseline and Placebo groups. Our findings suggest that intake of high levels of gamma-TT may reduce the absorption of the alpha isoform, similar to the competition between alpha- and gamma-tocopherols for hepatic secretion (56). The competition of oxidative enzymes for alpha-TT degradation rather than absorption by gamma-TT led to a decrease in alpha-CEHC sulfate.

Bone and skin contain large amounts of proline/hydroxyproline, and bone is a mixture of hydroxylapatite and collagen (57). Bone mineralization is a complex process in which type 1 collagen and associated non-collagenous proteins, such as proteoglycans and glycoproteins, interact closely with inorganic calcium and phosphate ions to control the precipitation of nanosized, non-stoichiometric hydroxyapatite within the organic matrix of a tissue (57). Proline-hydroxyproline (pro-hydroxy-pro), and C-glycosyltryptophan are derived from the degradation of proteins bearing post-translationally modified tryptophan and proline residues which, in the case of hydroxyproline, is specific to the collagen family of proteins (58). Clinically, pro-hydroxy-pro has been considered as a classic marker of bone resorption/collagen degradation in urine (58). Miyamoto et al. reported that compared to normal BMD subjects, those with low BMD (osteopenia and osteoporosis) had significantly higher levels of hydroxyproline and lower levels of Gly-Gly and cystine, a dimer of cysteines connected by an S-S bond in serum (59). High levels of hydroxyproline in urine and plasma correlate with the increased osteocalcin secretion that is characteristic of high bone turnover (59). C-glycosyltryptophan, a secondary metabolite of tryptophan, is considered as a novel biomarker for aging and age-associated traits and negatively associated with hip bone mineral density (18). In the present study, the findings that both compounds (pro-hydroxy-pro (*p* = 0.0003) and C-glycosyltryptophan (*p* = 0.081) were decreased in the serum after 12 weeks of TT supplementation suggests that TT favors bone remodeling by a decreased bone resorption/collagen degradation in collagen turnover.

Proteoglycans are composed of glycosaminoglycans (GAGs) covalently linked to the core protein, with each GAG, such as chondroitin sulfate or heparin sulfate, consisting of repeating disaccharide units that contain a sulfated amino sugar, e.g., N-acetylglucosamine or N-acetylgalactosamine, linked to glucuronic acid or iduronic acid (60). In the present study, the observation of 11% lower N-acetylglucosamine/N-acetylgalactosamine (measured as an isobar, *p* = 0.09) and a 25% higher serum glucuronate levels further support that dietary TT favors bone metabolism in postmenopausal women, in part, by reducing the release of bone matrix (61). All findings of bone resorption-/collagen-associated metabolites in the TT-supplemented subjects described above is corroborated with our earlier findings of decreased bone resorption (a decrease in urine N-terminal telopeptides of type I collagen) and improved bone turnover rate (an increase in bone alkaline phosphatase/NTX ratio) (42).

In the present study, changes were observed in transmethylation and transsulfuration pathway biochemicals with significant decreases in serum betaine, dimethylglycine, methionine, and 5-methylthioadenosine (MTA) after 12 weeks of TT supplementation. Methionine can be used to synthesize cysteine, a limiting factor in glutathione synthesis, which is utilized in antioxidant defense (62). In this study for TT-supplemented subjects, lower betaine, dimethylglycine, methionine, and MTA levels were accompanied by lower gamma-glutamyl amino acids (e.g., gamma-glutamyltyrosine, gamma-glutamylleucine), which result from the enzyme gamma-glutamyl transferase (GGT, a biomarker associated with oxidative stress) that transfers the gamma-glutamyl moiety from glutathione to the acceptor amino acid. Gamma-TT administration has been shown to decrease GGT activity in cultured rat hepatocytes (63) and increase serum glutathione levels in rats (16). Our results suggest that 600mg TT supplementation contributes to a decrease in oxidative stress consistent with our previous finding that dietary TT supplementation suppressed oxidative stress in PMW, as shown by a statistically significance decrease in urine 8-hydroxy-2′-deoxyguanosine (an oxidative stress biomarker) (*P* < 0.001) in the 600mg TT group relative to the placebo group (42). Furthermore, TT may conserve methionine and cysteine amino acids, sulfur derivatives or lead to lower catabolism of these amino acids which are susceptible to oxidation by ROS.

Tyrosine and tryptophan metabolite levels were lower after TT supplementation (Figure 4). Changes in tyrosine were associated with lower levels of tyrosine, 4-hydroxyphenylpyruvate, and 3-(4-hydroxyphenyl) lactate (HPLA) in subjects given 600mg TT compared to baseline after 12 weeks. Also, HPLA was higher in the Placebo group after 12 weeks from baseline. Kynurenine is produced by indoleamine 2,3-dioxygenase from tryptophan in response to inflammatory stimuli, e.g., TNF-α and IFN-γ, and has an anti-inflammatory function, serving as a brake to immune responses (64). The decreases in the levels of kynurenine and its downstream metabolite quinolinate are consistent with reported anti-inflammatory activities of TT (65). In this study, lower kynurenine and its downstream metabolite quinolinate, detected after 12 weeks of TT supplementation, may reflect decreased inflammatory signaling in the TT-supplemented subjects. The effects of annatto-TT on inflammatory markers have been reported by Qureshi et al., that annatto-extracted TT significantly suppresses the inflammatory production (i.e., resistin, IL-1δ, IL-12, TNF-α, IFN-δ, IL-6, c-reactive protein) in hypercholesterolemic subjects (66).

Knudsen et al. reported that butyrate has been shown to be a potent metabolic and inflammatory modulator in the development of non-alcoholic fatty liver disease. Phenylacetic acid, imidazole propionate, and HPLA have been identified as potential inducers of steatosis and hepatic inflammation, whereas indolic compounds (indole and indole-3-acetate) seem to preserve liver integrity (67). Caussy et al. demonstrated that HPLA level was significantly correlated with the abundance of several gut-microbiome species, belonging only to Firmicutes, Bacteroidetes, and Proteobacteria phyla, and reportedly associated with advanced fibrosis in non-alcoholic fatty liver disease (68). In the present study, both HPLA and 3-indoxyl sulfate, microbiome-derived compounds, showed changes due to TT and the lower level of HPLA and higher 3-indoxyl sulfate may suggest alterations in the gut microbiome activity and composition associated with TT supplementation. We previously reported that TT supplementation led to a metabolically favorable gut microbiome, as shown by the decreased relative abundance of Verrucomicrobia and increased relative abundance of Firmicutes in TT-supplemented obese mice (12).

Steroid hormones are synthesized from cholesterol in the adrenal glands and can undergo esterification with sulfate in the liver and brain (69). In addition to controlling inflammation and immune response, steroid hormones help control metabolism and physiology (69). In the present study, TT supplementation resulted in lower levels of steroid hormones, such as DHEA and progesterone; however, the impact on cholesterol level was only marginal (Figure 5). Since this study was not originally designed to evaluate blood lipids such as cholesterol and triglycerides, the heterogeneity in the lipid levels of subjects may have resulted in a ceiling effect for lowering cholesterol. However, TT has been reported to bind to estrogen receptor beta (ERbeta) (40) and to steroid and xenobiotic receptor (SXR) (70) to modulate the expression of target genes, including P450 family enzymes. In the current study, the changes in steroid hormones may be influenced by TT to alter levels of DHEA and progesterone. The effects of TT administration on steroid hormones are novel and should be confirmed with additional metabolic and physiologic studies.

Lysophosphocholines (LPCs) were postulated to promote inflammatory effects until recent opposing evidence (71). Specifically, LPCs have been identified to signal through G2A receptor present in regulatory T-cells to exert immunosuppressive functions (72). In the current study, there were higher metabolite levels of lysophospholipids in both 600mg TT and Placebo groups. These are phospholipids-derived degradation products that are formed when lipases remove fatty acid moieties from Sn-1 (PLA_1_) and Sn-2 (PLA_2_) position of phospholipids (73). The differences in the various lysophospholids metabolites after supplementation with TT may indicate biomembrane remodeling and their anti-inflammatory effects. The TT accumulates in biomembranes and remodels the signaling lysophospholipids. Thus, TT can change lysophospholipids and related signaling molecules. In the present study, the finding of higher levels of LPCs (e.g., 2-palmitoyl-GPC, 1-palmitoleoyl-GPC) in serum after TT supplementation could also suggest an increase in PLA_2_, ultimately resulting in altered lymphocyte function. This observation may be important since literature indicates that B- and T-cells are critical for the preservation of bone homeostasis and bone mass (74).

Carnitine (levocarnitine) is a naturally occurring compound found in all mammalian species. An important biological function of L-carnitine is in the transport of fatty acids into the mitochondria for subsequent β-oxidation, a process which results in the esterification of L-carnitine to form acylcarnitine derivatives. As such, the endogenous carnitine pool is comprised of L-carnitine and various short-, medium- and long-chain acylcarnitines. The physiological importance of L-carnitine and its obligatory role in the mitochondrial metabolism of fatty acids have been clearly established (75). It has been noted that the acylcarnitines are in equilibrium with their CoA ester forms, and thus the pattern may also reflect these pools (75). When cellular free fatty acids are in excess, the cells are able to utilize them in β-oxidation or complex lipid assembly. Acylcarnitines can cross the cellular membrane to be exported to the bloodstream (75). In the present study, the serum levels of acetylcarnitine (the shortest form of acylcarnitines) were lower in TT-supplemented subjects, indicating alterations in tissue fatty acid β-oxidation.

The effects of TT on fatty acid synthesis/metabolism are intriguing, and have been observed previously. Allen et al. reported that TT increased carnitine palmitoyltransferase (CPT) 1A and CPT2 (73). Gamma-TT reduced the mRNA expression of fatty acid synthase, stearoyl CoA desaturase 1 (SCD1), and sterol regulatory element-binding transcription factor-1 (SREBF-1), a transcriptional factor of genes in fatty acid synthesis, but increased the mRNA expression of carnitine palmitoyl transferase 1A (CPT1A) in mouse Hepa 1-6 hepatoma cells (76). Similar effects of TT on FAS and CPT1A mRNA and proteins were observed in HepG2 cells (77). In addition, studies demonstrated that gamma-TT reduced triglyceride via down-regulating various lipogenic enzymes such as fatty acid synthase, SREBF1, SCD1, and CPT-1A in mouse liver (76). Muto et al. further demonstrated that gamma-TT reduced the mRNA expression of SREBP-1c but increased the mRNA expression of CPT1A in hepatocytes obtained by perfusion from a 7-week-old rat (78).

Further, it appears that TT resulted in higher levels of PUFA, which might be related to greater elongation (2 carbon units malonyl ACP). Moreover, TT led to higher levels of lysophospholipids. Lysophospholipids are bioactive signaling lipids that are generated from phospholipase-mediated hydrolyzation of membrane lipids. Other findings show that a placebo group (860 mg of olive oil per day) had lower saturated and monosaturated metabolite levels, and PUFA metabolism consistent with obese mice (79).

In conclusion, our global metabolomic study of postmenopausal women with low bone mass found that the 12-week TT supplementation altered a number of biochemicals representing metabolism related to redox homeostasis, bioactive lipids, amino acid metabolism, steroid hormones synthesis, and bone degradation/collagen turnover. Changes in redox homeostasis manifested in transmethylation and transsulfuration pathway biochemicals could stem from the antioxidant activity of TT. Differences in compounds associated with bone degradation/collagen turnover indicate that, consistent with our previous findings, TT affects bone metabolism and collagen synthesis/degradation. Further, TT supplementation may conserve methionine and cysteine amino acids, sulfur derivatives or lead to a lower catabolism of these amino acids. Changes in tyrosine and tryptophan metabolites would suggest changes in the gut microbiome activity. Finally, differences in steroid hormones may point toward yet unreported effects of TT on the expression of steroid biosynthetic enzymes. As the members of the vitamin E family gaining more recent interests, tocotrienols have demonstrated broad biological effects on oxidative stress, inflammation, cell proliferation, adipogenesis, osteoclastogenesis, and osteoblastogenesis, and lipid metabolism. Our findings provide evidence to pursue future studies that would afford a mechanistic understanding of the nutritional values and health benefits of TT on bone remodeling and osteoblast functions.

## Data Availability Statement

The raw data supporting the conclusions of this article will be made available by the authors, without undue reservation.

## Ethics Statement

The studies involving human participants were reviewed and approved by Texas Tech University Health Sciences Center Institutional Review Board. The patients/participants provided their written informed consent to participate in this study.

## Author Contributions

CLS, HM, DMD, and BAW: conceptualization and writing—review and editing. CLS and BAW: methodology and writing—original draft preparation. CLS: supervision, project administration, and funding acquisition. All authors have read and agreed to the published version of the manuscript.

## Funding

Department of Pathology, Texas Tech University Health Sciences Center, Lubbock, TX, provided fund for metabolomics analysis. American River Nutrition, LLC., Hadley, MA supplied the tocotrienol supplement and funded this clinical trial. The funder was not involved in the study design, collection, analysis, interpretation of data, the writing of this article, or the decision to submit it for publication.

## Author Disclaimer

The content of this manuscript is solely the responsibility of the authors and does not necessarily represent the official views of American River Nutrition.

## Conflict of Interest

The authors declare that the research was conducted in the absence of any commercial or financial relationships that could be construed as a potential conflict of interest.

## Publisher's Note

All claims expressed in this article are solely those of the authors and do not necessarily represent those of their affiliated organizations, or those of the publisher, the editors and the reviewers. Any product that may be evaluated in this article, or claim that may be made by its manufacturer, is not guaranteed or endorsed by the publisher.
